# Supplementary material for: Healthy Food Policies Documented in University Food Service Contracts
Source: Int J Environ Res Public Health. 2023 Aug 21;20(16):6617. doi: 10.3390/ijerph20166617 (PMC10454280; doi:10.3390/ijerph20166617)
Supplement: Supplementary file 1 [file ijerph-20-06617-s001.zip › ijerph-2469201-supplementary.pdf]

UNC Charlotte Food Service Contract
Healthy Food Assessment Instructions

Instructions for assessing adherence to NC Healthy Food Guidelines at Institutions of Higher Learning that Partner with a Food Service Contractor

1. Request the university-food service contract. If the university is public, the contract can be requested through the legal department as a public records request.
2. Once the contract is obtained, complete the “Initial” worksheet of Food Service Contract Healthy Food Assessment* spreadsheet.
3. Copy row 2 of the “Initial” worksheet and paste the contents into row 2 of the “Feedback” worksheet.
4. Identify the food service director at the university.
5. Send the Food Service Contract Healthy Food Assessment to the food service director.
6. Ask the food service director to complete the “Feedback” worksheet of the Food Environment Summary spreadsheet.
7. Ask the food service director to return the spreadsheet to you.

*Completing the Food Service Contract Healthy Food Assessment

1. In column A “School”, type in the name of the institution.
2. In column B “Company”, enter the name of the Food Service Contractor.
3. In column C “Food Service Contract Date”, enter the date the food service contract was signed by the food service contractor.
4. In column D “Relevant contract page numbers”, enter the contract page numbers where the information needed to complete the assessment were found.
5. In column E “Q1. Policy guidelines for service/sale of health foods,” enter a 0 if no policy guidelines for the service or sale of healthy foods is found in the contract and enter a 1 if a policy guideline for the service or sale of healthy foods is found in the contract.
6. In column F “Q2. Which food service venue(s) does the organization use to sell or serve food?”, the options are listed in yellow in rows 4-11.
7. Continue to complete columns G-AP in the same manner.
8. In column AQ, sum columns H-AP.

School	Q2. Company	Contract Date	Relevant page numbers	Q1. Policy guidelines for service/sale of healthy foods	Q2. Which food service venue(s) does the organization use to sell or serve food?)	Q3. Which of the following does the organization serve/sell? (check all that apply)	B1. Free access to chilled drinking water	B2. Low-fat milk with no added sugar	B3. 100% juice with no added sugar	B4. At least 50% beverages ≤40 cal/8 fl oz
Copy/Paste verbiage from contract on this line as a reference										
				1 – Yes	1 – Cafeterias/cafes	1 – Beverages: This category includes drinks such as water, milk, 100% juice, soft drinks, energy drinks, teas, and coffees.	1 – Yes	1 – Yes	1 – Yes	1 – Yes
				0 – No	2 – Vending machines	2 – Packaged Snacks: This category includes processed foods that are	0 – No	0 – No	0 – No	0 – No
					3 – Concession stands, snack bars,	3 – Prepared Foods: This category includes foods that are fresh, cleaned,		2 – Milk and/or	2 – Juice is never	
					4 – Distributive meal program					
					5 – Meals served to					
					6 – Hospital patient meals					
					7 – External catering					
					8 – Other: (text box given)					
	Feedback: 1-yes, 0-no									
	Use this row to justify response. Please let us know if you have a policy in place or if this is a practice but not a requirement									

P1. All packaged snacks contain ≤200mg sodium	P2. All packaged snacks have 0 grams trans fat	P3. All packaged snacks at least one: 1st ingredient fruit/vegetable/dairy/protein; at least 1/4 fruit/vegetable; at least 50% whole grain	P4. All packaged snacks contain ≤200 calories per package	P5. All packaged snacks contain <10% of total calories from saturated fat	P6. All packaged snacks contain ≤35% of weight from total sugars	P7. All vending machines consistent w/ FDA Vending Machine Final Rule: Food Labeling/Calorie Labeling	Q4. At least 3 fruit options with no added sugars at all times	Q5. At least 3 non-fried vegetable options at all times	Q6. Seasonal fruits and vegetables	Q7. Half of total grains offered as "whole grain-rich" and remaining grains enriched	Q8. Variety of low-fat dairy products/alternatives such as milk, yogurt, cheese	Q9. Variety of non-fried protein foods such as seafood, lean meats and poultry, eggs, legunes, nuts, seeds, soy products	Q10. Protein foods from plants such as legumes, nuts, seeds, and soy products	Q11. Seafood at least two times per week	Q12. 25% of desserts contain ≤200 calories
1 – Yes	1 – Yes	1 – Yes	1 – Yes	1 – Yes	1 – Yes	1 – Yes	1 – Yes	1 – Yes	1 – Yes	1 – Yes	1 – Yes	1 – Yes	1 – Yes	1 – Yes	1 – Yes
0 – No	0 – No	0 – No	0 – No	0 – No	0 – No	0 – No	0 – No	0 – No	0 – No	0 – No	0 – No	0 – No	0 – No	0 – No	0 – No
						2 – The									

Q13. All meals contain ≤800mg sodium	Q14. All entrees contain ≤600mg sodium	Q15. All side items contain ≤230mg sodium	Q16. All foods free of partially hydrogenated oils	Q17. Provide calorie and nutrition information of standard menu items	Q18. Deep-friend options limited to no more than one choice per day	Q19. At least 10% spent on products originating in North Carolina	Q20. Organization signed up for NC 10% Campaign	Q21. Strategic placement to foster selection of healthier foods & beverages (flow paths, eye level, next to register, front of cold/hot entrees sections, within reach)	Q22. Product innovation and inclusion of healthier options as default choices	Q23. Price incentives and marketing strategies used to highlight healthier food and beverage items	Q24. Healthy portion sizes promoted by optimizing size of dishware and serving ware	Q25. Displays, decorations, and signage used to highlight healthier choices	Q26. Healthier options promoted via worksite wellness programs or other employee organizations	Q27. Changes to vendor contracts that support food service guidelines	Total number of categories addressed
															0
1 – Yes	1 – Yes	1 – Yes	1 – Yes	1 – Yes	1 – Yes	1 – Yes	1 – Yes	1 – Yes	1 – Yes	1 – Yes	1 – Yes	1 – Yes	1 – Yes	1 – Yes	
0 – No	0 – No	0 – No	0 – No	0 – No	0 – No	0 – No	0 – No	0 – No	0 – No	0 – No	0 – No	0 – No	0 – No	0 – No	
								2 – Not applicable			2 – Not applicable		2 – Not applicable		
															0
